# Audit of ADHD medication prescription and monitoring in intellectual disability services, Greater Glasgow & Clyde

**DOI:** 10.1192/bjo.2021.218

**Published:** 2021-06-18

**Authors:** Neha Bansal, Muzammil Hayat

**Affiliations:** NHS Greater Glasgow and Clyde

## Abstract

**Aims:**

Studies have shown that people with intellectual disability (ID) show a greater severity of attention deficit hyperactivity disorder (ADHD) symptoms and atypical presentation, as well as having a greater risk of developing comorbidities, such as challenging behaviour, anxiety, tic disorders and sleep problems. It is estimated that 1.5% of patients with ID will have a clinical diagnosis of ADHD.

The aim of this audit was to find whether individuals with ID and ADHD, who are prescribed medication for ADHD are adequately monitored and reviewed in accordance with the ADHD medication prescription guidance by NICE and the Royal College of Psychiatrists (RCPsych).

**Method:**

This audit looked at ADHD medication prescription for the ID population within Greater Glasgow & Clyde NHS. This is the 6th audit cycle where electronic records (EMIS) were analysed between 28/9/19 to 09/10/20. (The 5th cycle data collection period ended on 28/9/19). We collected data on all patients aged over 18 years.

An audit tool was developed to find whether the following were documented; patient demographics, physical health monitoring, symptom severity, medication dosage, side effects, need for ongoing treatment and frequency of review. 100% of patients should have all components on the ADHD audit tool documented, as per NICE/ RCPsych prescription guidance.

**Result:**

32 patients were identified as being diagnosed with ADHD prescribed medication. One patient was impacted by the COVID-19 pandemic which meant that the required monitoring was not fully carried out. The age ranged from 18 to 56 years. 75% had mild intellectual disability, 19% had moderate and 6% had severe, with no cases of profound intellectual disability. Blood Pressure/pulse was recorded in 84% of patients. Height/weight/ BMI was recorded in 81% of patients. 97% of patients had ADHD symptom severity, medication dosage, side effects, need for ongoing treatment and frequency of review recorded.

**Conclusion:**

There is further scope for improvement in the monitoring and documentation of physical health observations, however there was a significant improvement compared to the previous cycle of the audit. Other aspects of monitoring and documentation appear to be recorded in almost 100% of patients. This finding emphasises the challenges of physical health monitoring and compliance in psychiatry as a whole. We need to continue to encourage awareness and education around the physical health risks to our patients, not only due to their comorbidities but also as a result of the psychotropic medications we prescribe them.

